# Did You Care for the Orchids This Week? Outcomes of Person-Centered Cognitive Therapy for Parkinson’s Disease

**DOI:** 10.1093/geroni/igaf122.2289

**Published:** 2025-12-31

**Authors:** Magdalen Balz

**Affiliations:** University of Massachusetts Boston, Boston, Massachusetts, United States

## Abstract

Motor deficits in people with Parkinson’s disease (PwPD) are recognized by healthcare professionals as a hallmark feature of the diagnosis. However, PwPD also experience cognitive decline that negatively impacts quality of life and communication. Cognitive treatment in PwPD has received less attention in clinical care and research. This intervention study aimed to increase functioning in PwPD by applying cognitive compensatory strategies in a telehealth, group therapy program, BrainFit. Based on schedule availability, 3 participants elected to begin BrainFit treatment immediately, and 4 participants elected to join the waitlist, non-treatment group. All participants (N = 7, 56-74 years old) completed a comprehensive cognitive assessment and executive functioning questionnaire. BrainFit participants generated measurable, personally relevant goals using Goal Attainment Scaling (GAS). Goals targeted diverse objectives, from increasing consistency with medication management to following a schedule to care for household orchids. All participants, including the non-treatment group, repeated assessments at post-treatment and again at 3-month post-treatment. At each assessment, BrainFit participants also measured their status with personal goals. Descriptive analyses were applied to examine pre, post, and 3-month post-treatment change. BrainFit participants improved in at least one personal goal from pre to post-treatment. Two out of 3 BrainFit participants maintained gains with a personal goal at 3-month post-treatment. Cognitive assessment results varied across all participants at pre, post, and 3-month post-treatment. This study was built on work from Spencer and colleagues (2020) and further supports cognitive compensatory strategy intervention for PwPD. Findings provide novel contributions by investigating a telehealth, group treatment modality for PwPD.

